# Anti-Gb3 Monoclonal Antibody Inhibits Angiogenesis and Tumor Development

**DOI:** 10.1371/journal.pone.0045423

**Published:** 2012-11-26

**Authors:** Ariane Desselle, Tanguy Chaumette, Marie-Hélène Gaugler, Denis Cochonneau, Julien Fleurence, Nolwenn Dubois, Philippe Hulin, Jacques Aubry, Stéphane Birklé, François Paris

**Affiliations:** 1 Inserm, UMR892, Nantes, France; 2 Université de Nantes, UFR des Sciences Pharmaceutiques et Biologiques, Nantes, France; 3 CNRS, UMR 6299, Nantes, France; 4 Institut de Radioprotection et de Sûreté Nucléaire, Fontenay-aux-Roses, France; 5 Inserm, SFR Santé UMS 016, Nantes, France; 6 Institut de Cancérologie de l'Ouest, Saint-Herblain, France; Ottawa Hospital Research Institute, Canada

## Abstract

Inhibiting the growth of tumor vasculature represents one of the relevant strategies against tumor progression. Between all the different pro-angiogenic molecular targets, plasma membrane glycosphingolipids have been under-investigated. In this present study, we explore the anti-angiogenic therapeutic advantage of a tumor immunotherapy targeting the globotriaosylceramide Gb3. In this purpose, a monoclonal antibody against Gb3, named 3E2 was developed and characterized. We first demonstrate that Gb3 is over-expressed in proliferative endothelial cells relative to quiescent cells. Then, we demonstrate that 3E2 inhibits endothelial cell proliferation *in vitro* by slowing endothelial cell proliferation and by increasing mitosis duration. Antibody 3E2 is further effective in inhibiting *ex vivo* angiogenesis in aorta ring assays. Moreover, 3E2 treatment inhibits NXS2 neuroblastoma development and liver metastases spreading in A/J mice. Immunohistology examination of the NXS2 metastases shows that only endothelial cells, but not cancer cells express Gb3. Finally, 3E2 treatment diminishes tumor vessels density, proving a specific therapeutic action of our monoclonal antibody to tumor vasculature. Our study demonstrates that Gb3 is a viable alternative target for immunotherapy and angiogenesis inhibition.

## Introduction

Anti-angiogenic therapy, including monoclonal antibodies (mAbs) and small molecule inhibitors, is considered a relevant approach that limits tumor progression by inhibiting tumor vasculature development [1]. Therapeutic mAbs alone or in combination with other drugs are already proposed in clinical use to target pro-angiogenic molecular pathways. After a period of benefit, however, those antibodies, such as Bevacizumab, fail to produce a lasting clinical response in most patients, due to compensatory mechanisms leading to adaptive resistance [2], giving evidence for an urgent need to develop new anti-angiogenic therapies targeting new molecular targets.

The anti-angiogenesis therapies mainly focus on blocking growth cytokines or related receptors, or over-expressed proteins anchored in the endothelial cell membrane. However, mAbs recognizing cell surface glycosphingolipids (GSLs) have recently been proven to be effective for adjunct cancer therapy targets [3]. GSLs are expressed mainly at the outer leaflet of the plasma membrane [4]. They consist of a hydrophobic ceramide membrane anchor and a hydrophilic cell surface-exposed oligosaccharide chain, accessible to cell surface recognition molecules, making them candidate targets for oncological applications [3]. In particular, globotriaosylceramide Gb3, a neutral GSL, has been identified as three different entities according to the cell type: the rare Pk blood group antigen on erythrocytes [5], CD77 differentiation antigen on germinal B lymphocytes [6], and the receptor of the bacterial toxin of the Shiga family, also called verotoxin on small intestine epithelial cells [7]. Gb3 is also found in the kidney glomerulus of the very young [8] and in several tumors, such as Burkitt's lymphoma [9], and colorectal, breast, pancreatic and ovarian carcinomas [10], [11], [12], [13], [14]. Gb3 is expressed not only in tumor cells, but also in the vasculature surrounding and within the tumor [15]. This latter finding may reflect an over-expression of Gb3, especially in angiogenic endothelial cells, which could be targeted by an anti-cancer agent.

Because Gb3 is a shiga toxin receptor, several engineered shiga toxin Gb3 ligands are currently under investigation as potential anti-cancer agents [16]. Its relevance for clinical trials, however, may be limited due to the toxin's residual immunogenicity [17]. Furthermore, a high prevalence of anti-shiga toxin antibodies was detected in healthy populations [18], which could be associated with population immunity to systemic shiga toxin-associated disease [18]. Due to these limitations, the use of mAbs specific to Gb3 would be more appropriate in patients.

In this study, we report the generation of a mouse IgM mAb specific for Gb3 named 3E2. We demonstrate that Gb3 is over-expressed in proliferating endothelial cells in culture. Furthermore this novel Mab 3E2 is shown to inhibit angiogenesis *in vitro* and *ex vivo* using a mouse aorta ring test. Finally, its ability to block tumor angiogenesis and subsequently tumor growth was evaluated successfully *in vivo* in a syngeneic animal model. Thus, the use of anti-Gb3 mAbs could represent new therapeutic strategies for anti-angiogenic therapies.

## Materials and Methods

### Cell lines, antibodies and lipids

Human microvascular endothelial HMEC-1 cells and murine neuroblastoma NXS2 cells respectively provided by F. J. Candal (Center for Disease Control, Atlanta, USA) and by Pr. Lode (Universitätsklinikum Greifswald, Germany) were grown as previously described [19], [20]. Human neuroblastoma IMR32 and Burkitt's lymphoma Raji cells were grown in RPMI 1640 with 10% fetal Calf serum as described [21], [22]. T84 cells were grown in DMEM∶F12 (1∶1, Gibco) supplemented with 10% heat-inactivated FBS as described [23], The rat anti-Gb3 mAb (clone 38.13; Beckman Coulter) and the GD2 mAb 14G2a (BD Biosciences) and their isotypic controls (clone 11E10; Beckman Coulter) were diluted in PBS. Rat brain gangliosides extract, the neutral GSLs mixture and purified-Gb3 were purchased from Matreya LLC. Sphingosine-1-phosphate (S1P; Biomol) was rehydrated in PET diluent as previously described [19].

### Hybridoma generation

Murine 3E2 mAb (IgM, κ) specific to Gb3 was established in our laboratory. 6-week old Balb/c mice (Janvier) were immunized by intra-peritoneal injection of proliferating primary human lung microvascular endothelial cells HMVEC-L (Lonza) grown in presence of human colon T84 adenocarcinoma cells (ATCC) in culture condition previously described [23]. Mice were immunized with 5 doses of 2.5×10^6^ HMVEC-L cells every 10 weeks and one booster shot of 1.5×10^6^ cells. Splenocytes were fused with myeloma cells SP2/O-Ag14 using polyethylene glycol. Hybridoma fusions were screened on either proliferating or non-proliferating endothelial cells. After limiting dilution, 3E2 mAb was affinity-purified from culture supernatants by HiTrap Protein L column (GE Healthcare).

### Glycolipids extraction HPTLC analysis and immunostaining

Glycolipids were extracted and analyzed according to Ladish *et al.* [24]. The recovered glycolipids (2.5 mg of protein equivalent per sample) were dissolved in a 1∶2 v/v chloroform-methanol solution and separated on a 55∶45∶10 v/v/v chloroform-methanol-0.2% aqueous CaCl_2_ solution on thin-layer chromatography plates (HPTLC; Merck). Glycolipids bands detected after orcinol spray were quantified by densitometry (ImageQuant, GE Healthcare). GSLs were identified by the nomenclature of Svennerholm [25]. Then, TLC plates were fixed by 0.1% polyisobutylmethacrylate in hexane, saturated with 1% PBS-BSA and overlaid with antibodies overnight at 4°C (5 µg/ml in 0.1% PBS/BSA). mAb binding was detected by 4-chloro-1-naphtol solution (Sigma) after incubation with biotinylated anti-mouse immunoglobulin (Invitrogen; 1∶2,000), and steptavidin-horseradish peroxidase complex (Beckman Coulter; 1∶2,000) for 1 h each.

### ^3^H-thymidine incorporation

16 h after treatment, HMEC-1 were incubated with accurate medium containing ^3^H-thymidine (1 µCi/ml) for 8 h. Cells were then processed as described [19].

### Flow cytometry

To detect cell-surface antigens, 2×10^5^ cells were incubated at 4°C with 3E2 or 11E10 (40 µg/ml) for 60 min, pelleted at 300 g for 1 min and fixed for 15 min with 4% paraformaldehyde. Antibody binding was detected by stepwise incubation at 4°C with biotinylated chain-specific anti-mouse IgM (Jackson Immunoresearch; 1∶500), then by a FITC-steptavidin complex (Beckman Coulter; 1∶1000) for 30 min each. Cell fluorescence (10^4^ cells) was analyzed in a FACScan running Cell Quest Pro (BD Bioscience).

### Real-time quantitative PCR

Cellular RNAs from HMEC-1 cells were isolated using the Nucleospin RNA II kit (Macherey-Nagel) according to the manufacturer's instruction. One µg of total RNA was Oligo(dT) primed and first-strand cDNA synthesis was performed according to the manufacturer's guidelines (Super Script™ III RNase H Reverse Transcriptase, Invitrogen). mRNA expression quantifications expression were analysed by real-time quantitative PCR on 50 ng of cDNA in 50 µl SYBR Green PCR Master Mix mix (Roche) incubated with the Gb3 synthase primers described [26]. Glyceraldehyde-3-phosphate dehydrogenase was chosen as endogenous reference for normalisation. Relative gene expression was calculated using the comparative Ct method [27].

### Immunocytology

HMEC-1 seeded on coverslips were incubated at 4°C with 3E2 (40 µg/ml) for 45 min, and fixed for 15 min with 4% paraformaldehyde. The mAb binding was stepwise incubated at 4°C with biotinylated anti-mouse IgM (Jackson Immunoresearch; 1∶400) for 30 min, FITC-steptavidin complex (Invitrogen; 1∶400), and Draq5 counterstain (1∶1,000; Biostatus) for 15 min. and detected under confocal microscopy (TCS-SP1, Leica).

### Cell viability by MTT

2×10^4^/cm^2^ HMEC-1, NXS2 or RAJI cells were plated in 24-wells, 12 h before incubation of the 3E2 or its isotypic mAb at various concentrations. After 24 h, 166 mg/ml of the 3-(4,5-dimethylthiazol-2-yl)-2,5 diphenyltetrazolium bromide solution (Roche) were added for 4 h at 37°C. Color was revealed after overnight incubation at 37°C with 100 µl of 10% SDS in 0.01 M HCl and quantify at 570 nm on a Multiskan reader (Thermo Electron).

### Western Blot

After lysis, 3E2-or IgM-treated HMEC-1 proteins were separated by SDS-PAGE and transferred to ImmobilonP membrane (Millipore). The membrane was hybridized with anti-bodies of interest diluted at 1/1,000 for phospho-AKT (clone 9271; Cell Signaling), 1/1,000 for phospho-ERK (clone 9106; Cell Signaling), and 1/2,500 for actin (Santa Cruz).

### Time-lapse assessment

2×10^4^/cm^2^ HMEC-1 in 24-wells maintained at 37°C, 5% CO_2_ were incubated with 3E2 or control at 20 µg/ml. Digital images were taken every 10 min over 24 h under a time-lapse DMI6000B microscopy (Leica). Number of cells, mitosis index and cell detachment have been summed over the 24 h on 3 different 10× magnified microscopy shots per well.

### Apoptotic assays

2×10^4^/cm^2^ HMEC-1 were treated with either 3E2 or its IgM control 11E10 at 20 µg/ml for 24 h before fixation and cell death analysis. Cell death was detected by morphology assay after Hoechst coloration or by flow cytometry analysis of the sub-G1 cell population after propidium iodide (PI) as described [28], [29].

### Aortic ring assay

8-week old male C_57_BL/6 mice aortas were isolated as described [30]. Aorta rings were treated with either 3E2 or IgM control for 5 d. Then, pictures were shot at 100× magnification and a score based on the number, the length and the density of vessels, ranging 0 (no sprouting) to 5, was attributed by five independent panelists.

### Metastatic and transplanted Murine tumor models

Six to eight week male A/J mice (Harlan) were studied following accordance with European Communities Council for the care of laboratory animals (86/609/EEC). Experimental hepatic metastases were induced by tail vein injection of 2.5×10^5^ NXS2 on day 0 as described by Lode *et al.* [20]. Mice were treated with mAb *i.v.* injections on day 1, 2, 7 and 8 for 3E2 (200 µg in 150 µl PBS). Negative control mice received PBS only or control Ig. Positive control mice received anti-GD2 14G2a (100 µg in 100 µl PBS) on day 1 and 2. Animals were sacrificed on day 28, and the number of liver metastases was evaluated.

Established NXS2 tumors were induced by subcutaneous injection of 10^6^ NXS2 cells in the right flank of A/J mice. When the tumor reached a volume of 100 mm^3^, mice were treated with mAb *i.v.* injections on day 0 and 3 for 3E2 (500 µg in 200 µl PBS). Negative control groups received control 11E10 IgM or PBS. Tumor size was measured every day with a digital caliper, and volume was calculated by applying the formula: volume (mm^3^) = length×width^2^×0,5. The end point of these studies was determined by both tumor size (>2000 mm^3^) and the condition and behavior of the animal. Morbidity is calculated when tumor reached the volume of 300 mm^3^.

### Immunohistology

Frozen tumors or healthy organs sections of 5 µm were stained 90 min with rat anti-mouse CD31 (Millipore; 1∶50) and biotinylated 3E2 or its isotypic control (40 µg/ml) by EZ-Link Sulfo-NHS-LC-Biotinylation Kit (Thermo Scientific). Staining was revealed by 90 min of incubation with Alexa 488-conjugated goat anti-rat Ab (Invitrogen; 1∶400) for CD31 and Alexa 568-conjugated streptavidin (Invitrogen; 1∶200) for Gb3. Samples were counterstained with DAPI. Pictures were observed under an A1 confocal fluorescence microscope (Nikon). Colocalization between CD31 and Gb3 were processed by ImageJ with a median filter. Then, a threshold was applied to each channel of fluorescence. The binary resulting masks from the green and red channels were merged by the “colocalization” analysis processing, which shows only the common pixels to both channels.

### Blood vessel density

Frozen tumor sections from the different treatments and time points were stained with anti-CD31 antibody and counterstained with 5 µM Draq5 as detailed above. The slides were examined using an Axiovert 200 microscope (Carl Zeiss) with a 20× apochromat. images were collected at an optical section thickness of 0.4 µm through the entire cell using the Apotome slider on the Axiovert 200. CD31 Fluorescence was quantified by ImageJ for each condition. Mosaic images made of 24 fields (6×4) were jointed to obtain a larger image.

### Scatchard analysis

Scatchard binding assays were performed, as described [21] on iodine-125-labeled 3E2 with 10^6^ cells. Cell-bound radioactivity was separately measured using a gamma-counter (Wallac). The binding data were analyzed according to a one-site equilibrium binding equation.

### Statistic analysis

Statistical analysis was performed using GraphPad Prism software for Student's t test, with 95% confidence estimations or 2 ways-ANOVA tests. Actuarial survival and morbidity was calculated by Kaplan-Meier product limit estimation and statistical significance in survival by the Mantel log-rank test.

## Results

### 3E2 antibody targets Gb3

To generate mAbs against Gb3 with potent anti-angiogenic activity, we immunized Balb/c mice with proliferating primary microvascular endothelial HMVEC cells activated by T84 adenocarcinoma cells in a co-culture system (see Materials and Methods). After ELISA- and immunofluorescence-based screening, we established 6 hybridoma clones with reactivity to Gb3. 3E2 hybridoma clone with IgM isotype was selected for its strong binding properties to Gb3 (data not shown).

**Figure 1:**
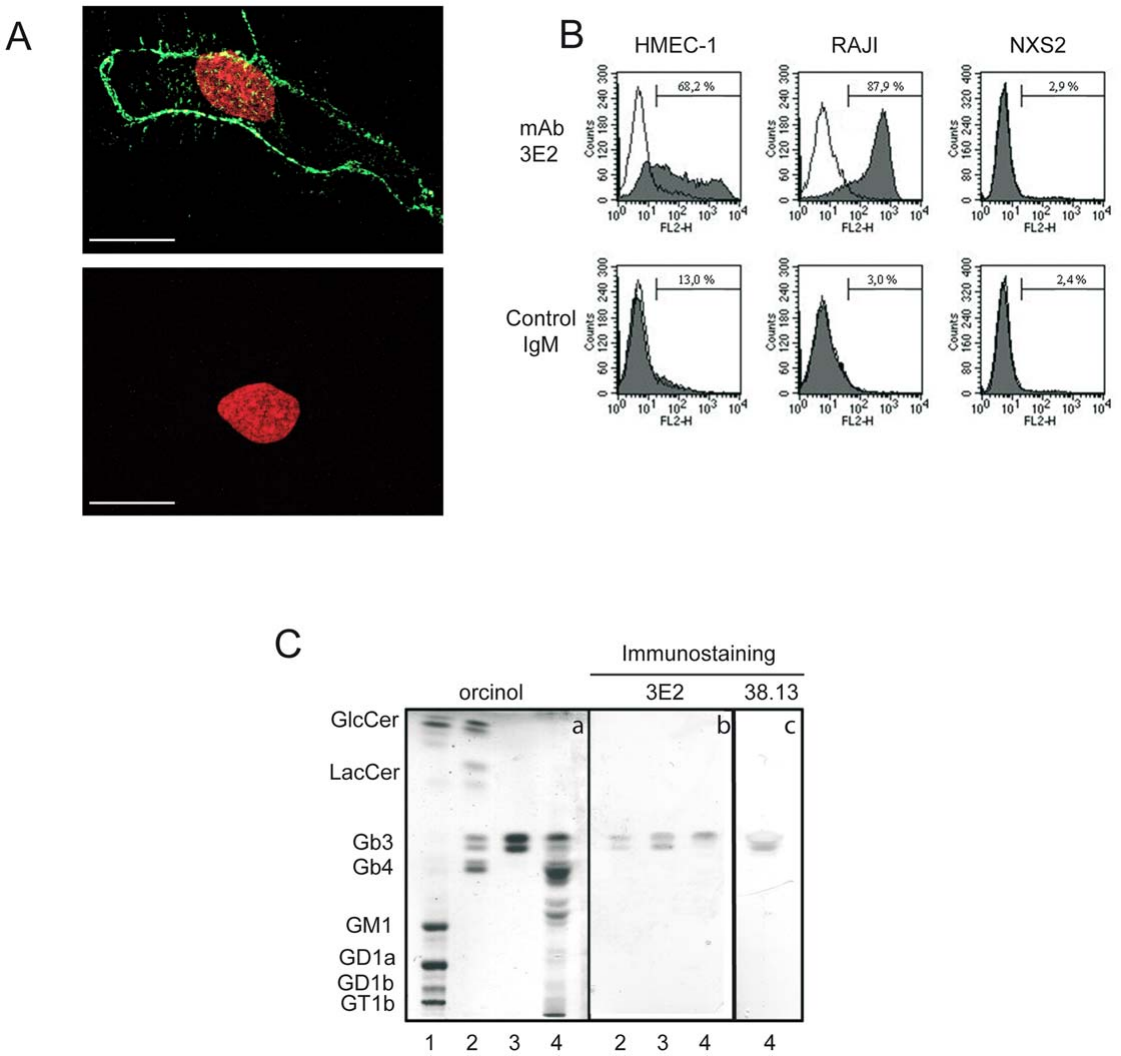
3E2 targets Gb3 on the endothelial cell membrane. A) Gb3 immunolocalization in HMEC-1 by FITC-conjugated 3E2 (green; upper panel) *vs.* control isotypic 11E10 IgM (lower panel) and counterstaining with Draq5. Scale bar represent 20 µm. B) Percentage of Gb3-positive cells by Facs analysis after hybridization of HMEC-1, RAJI and NXS2 cells with using 3E2 or isotypic control. C) GSLs profiles by orcinol staining after HPTLC (panel a) and Gb3 expression by immunohybridization using 3E2 (panel b) or commercially available 38.13 (panel c). Lane 1: rat brain gangliosides; lane 2: neutral GSLs mixture: lane 3, purified Gb3; lane 4: HMEC-1 glycolipids extract.

First, we show that 3E2, contrary to its isotypic control, binds on the cell membranes of proliferating HMEC-1 as shown by the green staining under confocal microscopy (Fig. 1A). By HPTLC, HMEC-1 is showing to express Gb3, such as RAJI (Fig. S1). We next demonstrated by FACS the exclusive binding of 3E2 to Gb3-expressing cell lines, HMEC-1 (3E2-positive cells: 68.2%; mean of fluorescence (MF): 568.3; Fig. 1B) and RAJI (3E2-positive cells: 87.9%; MF: 423.8), compared to the Gb3-negative cell line, NXS2 (2.9% of 3E2-positive cells; MF: 156.5). In the same conditions, control IgM do not bind to the Gb3-expressing cell lines (3E2-positive cells and MF for HMEC-1: 13%, 64.5; for RAJI: 3%, 82.5) confirming the 3E2 specificity. Finally, 3E2 binding activity against Gb3 was monitored by immunoTLC assay (Fig. 1C). Gangliosides mixture, neutral GSLs, pure Gb3 and HMEC-1 glycosphingolipids were loaded on silica TLC plate, then migrated in function of their hydrophobicity and molecular weight, as observed after orcinol staining (panel a). Hybridization with 3E2 showed the specific binding to a doublet on neutral GSLs, purified Gb3 and HMEC-1 lanes (panel b, lanes 2, 3 and 4), characterized as the Gb3 after orcinol staining (panel a, lane 3). No cross-reactivity of 3E2 is detected against lactosylceramide, glucosylceramide, GM3 or Gb4 as well with the remaining endothelial cells glycolipids (panel b, lanes 2 and 4). Gb3 specificity for the 3E2 was confirmed using another anti-Gb3 antibody the 38.13 mAb on HMEC-1, despite a potential discrepancy on the two bands affinity (panel c). Taken together with various immunological assays, our results demonstrated that 3E2 mAb specifically detect Gb3.

### Gb3 is over-expressed in proliferating endothelial cells

**Figure 2:**
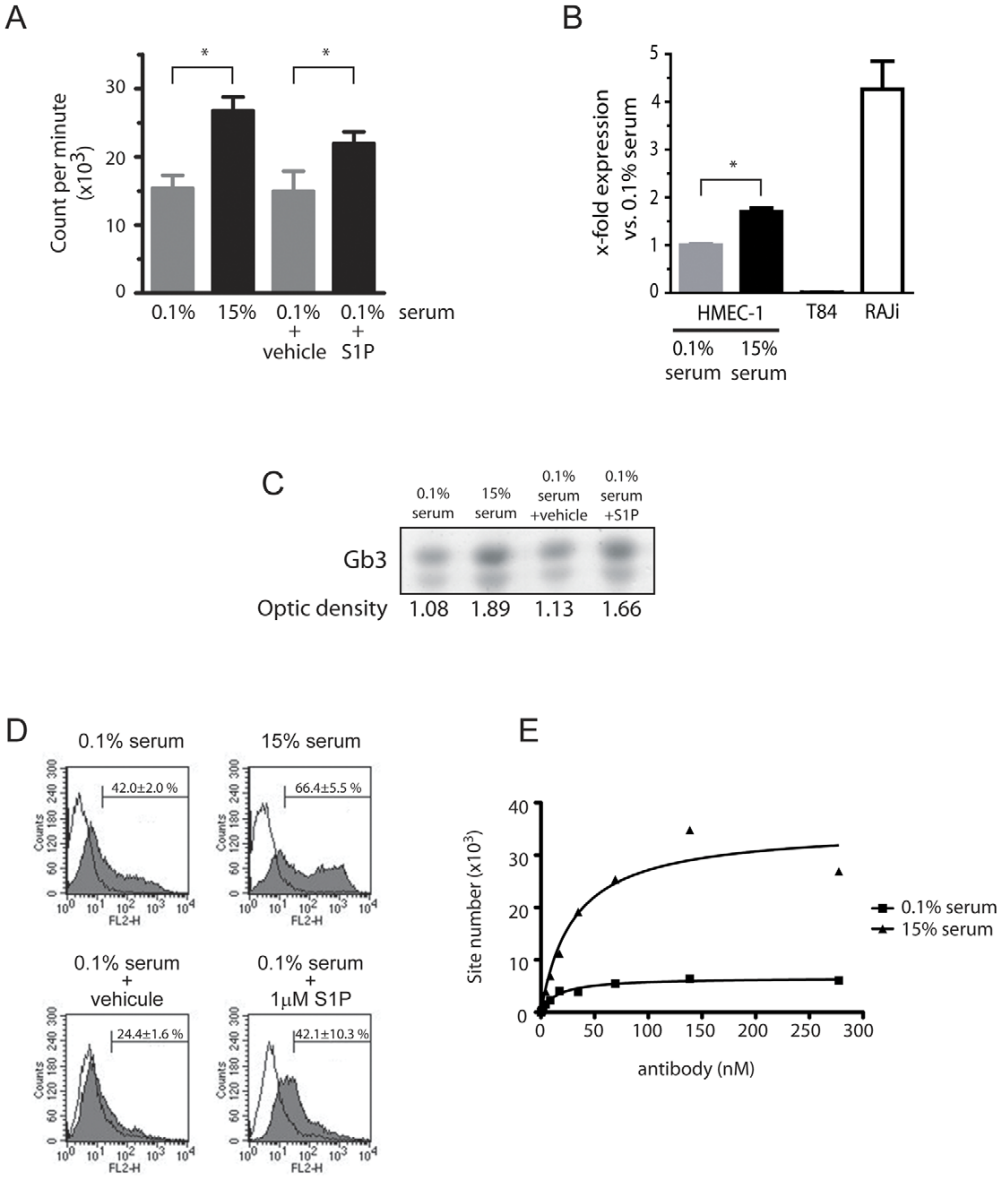
Gb3 is over-expressed in proliferating endothelial cells. HMEC-1 were treated with medium containing respectively 15% serum, 0.1% serum and 0.1% serum with 1 µM S1P or its vehicle. A) Proliferation assay by ^3^H-thymidine incorporation 24 h after treatment. (n = 3; mean±SD; *p≤0.05). B) RT-Q PCR 24 h after treatment (n = 6; mean±SEM; ns: p<0.01). C) Gb3 expression detected by 3E2 after immuno-HPLC. D) Gb3-positive HMEC-1 determines by Facs using 10 µg/ml of 3E2 (n = 3). E). Gb3 site number determined by Scatchard analysis using 3E2.

**Figure 3:**
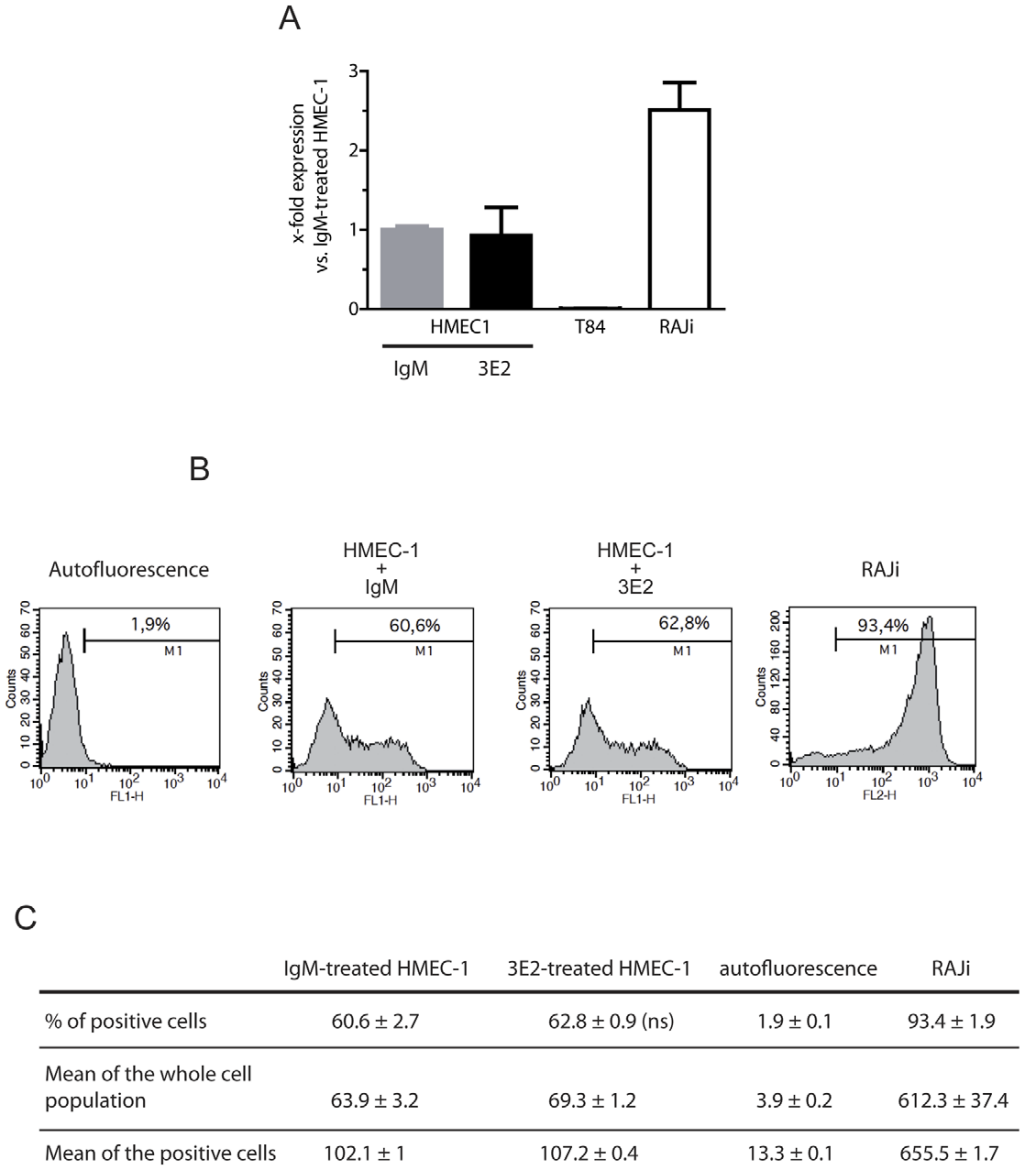
Gb3 is not modulated by 3E2 antibody treatment. HMEC-1 cells were treated with either 40 µg/ml 3E2- or IgM control-antibody. A) RT-Q PCR 24 h after treatment (n = 6; mean±SEM; ns: p>0.1). B) Gb3-positive HMEC-1 cells determined by Facs 24 h after treatment (n = 3). C) Table analysis (% of positive cells, means) from Fig. 3 B (ns>0.1).

We next analyzed the amount of Gb3 molecules at the cell surface of either proliferating or non-proliferating endothelial cells. As presented in Fig. 2A, lowering the serum concentration to 0.1% in the cell medium inhibits by 42.5% the proliferation of HMEC-1 cells measured by thymidine incorporation assay (26756±2018 counts/min in 15% serum *vs.* 15379±1893 in 0.1% serum; p<0.05). On the other hand, adding 1 mM of pro-angiogenic S1P to low serum medium increase by 32% the cell proliferation (14941±1719 counts/min in 0.1% serum+vehicle vs. 21959±1719 in 0.1% serum+S1P; p<0.05). Then, Gb3 synthase expression was evaluated by quantify the mRNA level by RT-QPCR in function of the HMEC-1 proliferative status (figure 2B). We next quantify the Gb3 synthase mRNA level by RT-QPCR in function according to the HMEC-1 cells proliferative status (Fig. 2B). As expected, a high number of Gb3 synthase mRNA copies was detected in Raji cells, but not in T84 carcinoma cells. Interestingly, we found an 69% increase of Gb3 synthase mRNA copies in the proliferating HMEC-1 cells grown in the high serum medium compare to the same low dividing cells cultivated in the low serum medium (p<0.01). Because Gb3 synthase allows the production of Gb3 from lactosyl-ceramide, Gb3 expression was quantified by optic density (OD) after HPTLC in function of the HMEC-1 proliferative status (figure 2C). OD of Gb3 bands in endothelial cells cultured in pro-proliferative conditions were higher as compared to the low proliferating one (OD 75% higher in 15% serum vs. 0.1% serum; 47% higher in 0.1% serum+S1P vs. 0.1% serum). We confirmed the increase of Gb3 expression in proliferating endothelial cells by FACS (Fig. 2D). In fact, a larger number of Gb3 positive cells were stained by 3E2 in pro-proliferative as compared to pro-quiescent experimental conditions (66.4±6.1% for 15% serum vs. 42.0±2.0% for 0.1% serum; 42.1±10.3% for 0.1% serum+S1P vs. 24.4±1.6% for sham-control, p<0.05). MF providing the relative quantities of Gb3 in HMEC-1 for the different experimental conditions, increase by 2 in cells cultured in full medium as compared to those in deprivated medium (639.6±18.9% for 15% serum vs. 300.7±40.6% for 0.1% serum, p<0.01) and by 1.3 in S1P-treated cells as compared to its sham-control (204.6±32.4% for 0.1% serum+S1P vs. 159.7±2.5% for sham-control, p<0.05). Finally, the number of Gb3 molecules present at the cell surface was calculated by Scatchard analysis using I^125^-labelled 3E2 (Fig. 2E). Fast proliferating HMEC-1 express 8.5 fold more Gb3 molecules in their cell membrane than low proliferating endothelial cells (Gb3 sites per cell: for 15% serum: 1.7×10^6^, Kd value: 30.2 nM; for 0.1% serum 0.2×10^6^, Kd value: 30.2 nM). Altogether our results confirm that Gb3 is over-expressed by proliferating endothelial cells. We next analyzed if mAb 3E2 binding would modulate Gb3 expression in proliferating HMEC-1 cells. First, we demonstrated that 3E2 antibody binding to proliferating HMEC-1 cells did not change their Gb3 synthase mRNA expression level after 24 hours treatment (vs. IgM-treated cells p>0.1; Fig. 3A). In the same manner, cytometry analysis of Gb3 expression at the cell surface was not decreased by mAb 3E2 treatment (% of positive cells and means vs. control IgM-treated cells p>0.1; Fig. 3B and C) proving a potential sequential use of anti-Gb3 3E2 antibody.

### 3E2 inhibits endothelial cell proliferation and *ex vivo* angiogenesis

**Figure 4:**
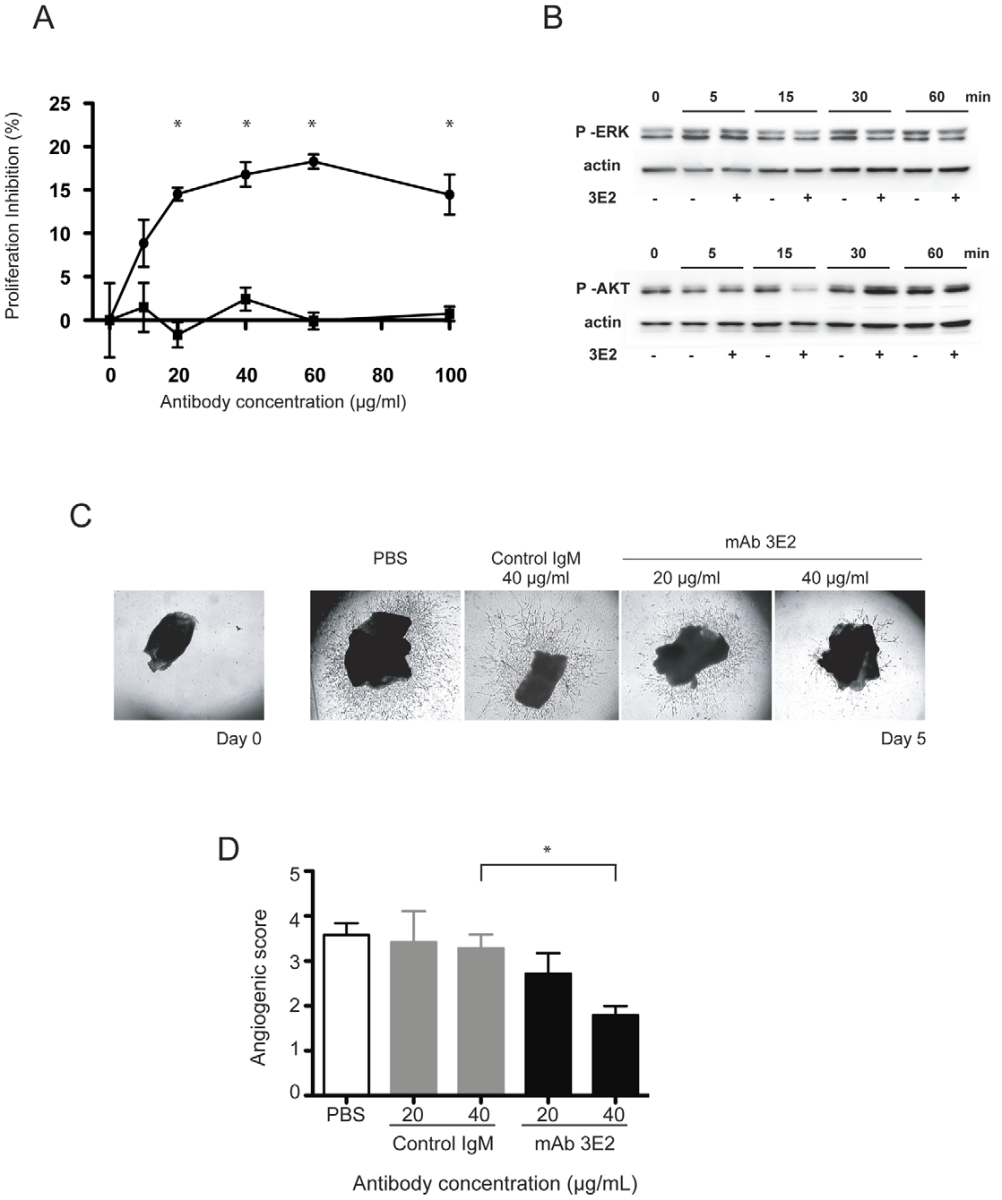
3E2 antibody inhibits *ex vivo* angiogenesis. A) Viability measured by MTT of HMEC-1 treated for 24 h with increasing concentration of 3E2 (▾) or isotypic control (▪) (n = 3; mean±SEM; *p<0.05). B) Western blot of phosphor-ERK and phosphor-AKT from cells treated with either 40 µg/ml 3E2 or IgM-control antibody (n = 3). C) Photographs of sprouting vessels from aorta 5 d post-treatment by an increasing dose of 3E2 (n = 3). D) Sprouting index from aorta rings 5 d post-treatment with 3E2 or isotypic control (n = 3, mean±SEM; *p<0.05).

To assess the anti-angiogenic properties of anti-Gb3 antibodies, we next measured the viability of HMEC-1 incubated with increasing doses of 3E2 for 24 h by MTT assay (Fig. 4A). 3E2, but not control IgM mAb, inhibits HMEC-1 viability in a dose dependant manner in the absence of complement to reach a maximum value of 18.3±0.8% when cells are incubated with 60 µg/ml of anti-Gb3 mAb (p<0.01 vs. IgM). Antibody 3E2 specific viability inhibitory effect was confirmed in the Gb3-expressing RAJI (data not shown), but not in Gb3-negative NXS2 (Fig. 4A). We next studied the anti-proliferating function of mAb 3E2 by investing its ability to modulate two of the ubiquitous molecular actors involved in angiogenesis and endothelial stress response [31]. In fact, protein analysis by western blot shows that activated forms of AKT and ERK were down regulated respectively 15 and 30 min after 3E2 antibody treatment (Fig. 4B). We further analyzed 3E2 anti-angiogenic properties in the *ex vivo* aorta ring assay (Fig. 4C and D). The sprouting formation of endothelial cells is clearly observed in aorta rings harvested from C_57_Bl/6 mice and cultured for 5 d in decomplemented medium. The sprouting formation is significantly reduced in 3E2-treated aortic rings in a dose dependent manner as shown by the decrease of the number of endothelial cell branches on the pictures (Fig. 4C) and by the sprouting index estimated double blind observation from 5 different persons (Fig. 4D). The specificity of 3E2 anti-angiogenic properties was demonstrated since IgM treatment in the same dose range does not inhibit the sprouting of aorta rings. These results evidence that anti-Gb3 mAb inhibits *in vitro* and *ex vivo* angiogenesis.

**Figure 5:**
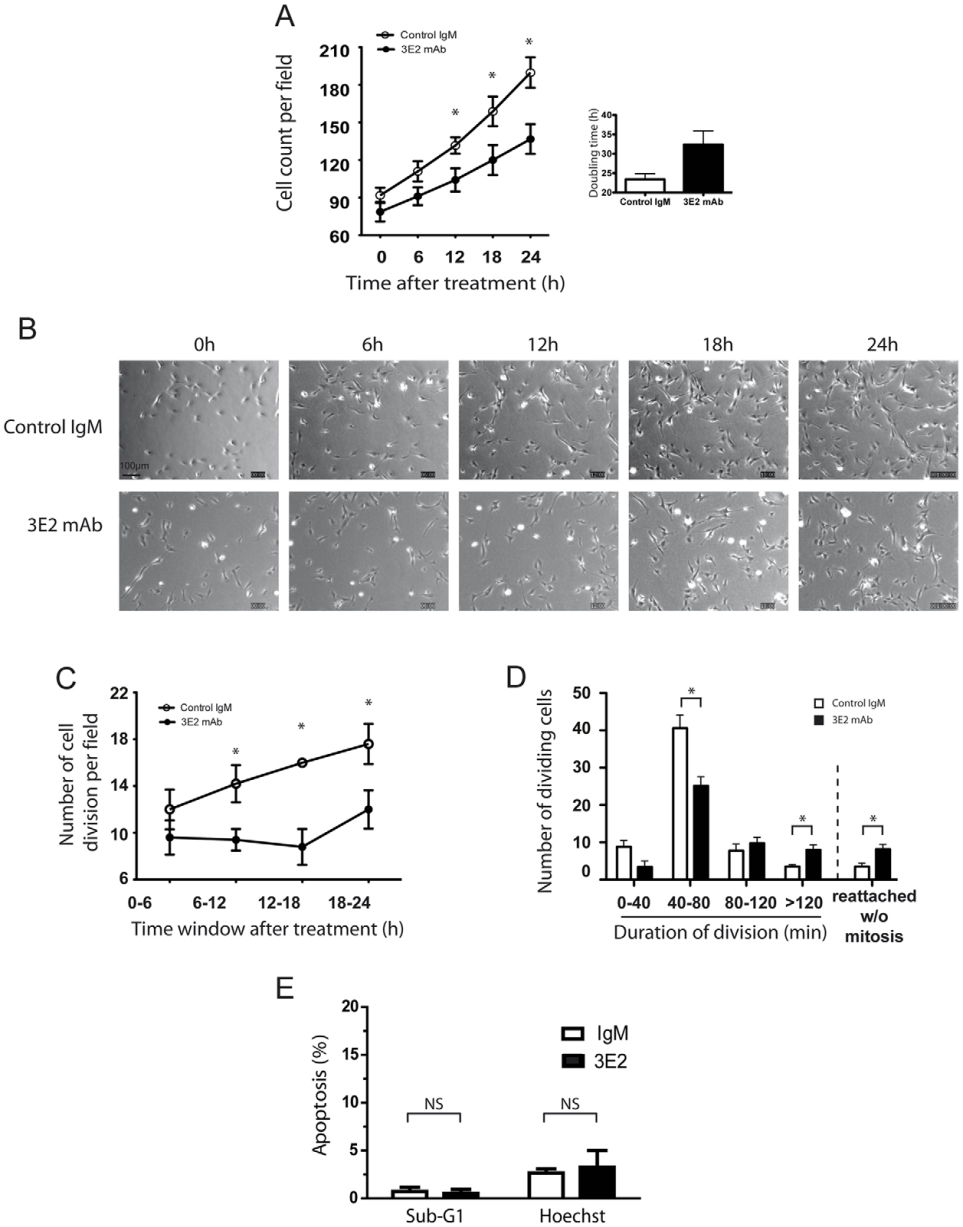
3E2 antibody inhibits endothelial cell proliferation. HMEC-1 cells are tracked by videomicroscopy up to 24 h after treatment by 3E2 or isotypic control. A) Cell number per field in function of time. Histograms show the mean doubling time (n = 6 for IgM and n = 9 for 3E2, mean±SEM; *p<0.05). B) Microphotographs of representative fields of HMEC-1 in function of time. Magnification 10×. C) Number of mitosis summed every 6 h for 24 h (n = 5, mean±SEM; *p<0.05). D) Duration of the mitosis (n = 5, mean±SEM; *p<0.05). E) Cell death quantification detected by sub-G1 and hoechst assays from 3E2- or IgM-treated HMEC-1 (n = 3; mean±SD; ns: p>0.1).

To better understand the mechanisms by which 3E2 inhibits *ex vivo* angiogenesis, we tracked 3E2- or control IgM-treated HMEC-1 for 24 h by time-lapse microscopy. We first studied endothelial cell proliferation by counting the number of cells per photomicrography field in function of time (Fig. 5A and B). On the contrary to control IgM, 3E2 treatment alters the endothelial cell expansion over the 24 h after treatment (mean doubling time for IgM: 23.4±1.5 h; for 3E2: 32.4±3.5 h; p<0.05; Fig. 5A). Then, the number of cell divisions was identified in 4 time windows of 6 h over the 24 h of the study. The mitotic phase was detected by morphological changes occurring when a flat adherent cell separates into 2 daughter cells (Fig. S2). The number of cell division increased in function of time when cells were incubated with the isotopic control (figure 5C). Treatment with 3E2 strongly inhibited cell division during the first 18 hours (35.8% decrease of cell division vs. control in the time windows 12–18 h post-treatment). Later, the cell division seems to restart in the 3E2 group, which could be over-passed by a repetitive incubation of our anti-Gb3 antibody (data not shown). We next estimated mitosis duration in the presence of either anti-Gb3 mAb or IgM. Mitosis of the majority of the cells occurred in 40 to 80 min in the control cells (Fig. 5D). Antibody 3E2 treatment reduced significantly by 38% the number of cells per field dividing in 40–80 min (25.2±2.3 in 3E2-treated cells vs. 40.6±3.5 in the IgM-treated cells; p<0.05) and also prolonged by 55% the mitosis over 120 min (8.0±1.3 cells in 3E2-treated sample vs. 3.6±0.5 cells in the IgM-treated sample; p<0.05) and increased by 56% the number of cells reattaching to the culture flask without mitosis (8.2±1.2 in 3E2-treated cells vs. 3.6±0.9 in IgM-treated cells; p<0.05). No cell death was detected during the 24 hours of the time lapse studies. We confirmed the lack of cytotoxic effect of the 3E2 antibody was observed using either flow cytometry analysis of the sub-G1 population by facs analysis or the percentage of the apoptotic morphology using Hoechst staining (p>0.1 vs. IgM–treated cells for both analyse; Fig. 5E). Taken all together, our results suggest 3E2 inhibits endothelial cell expansion by disturbing mitosis and cell proliferation without increasing cell toxicity.

### 3E2 inhibits NXS2 metastases spreading

**Figure 6:**
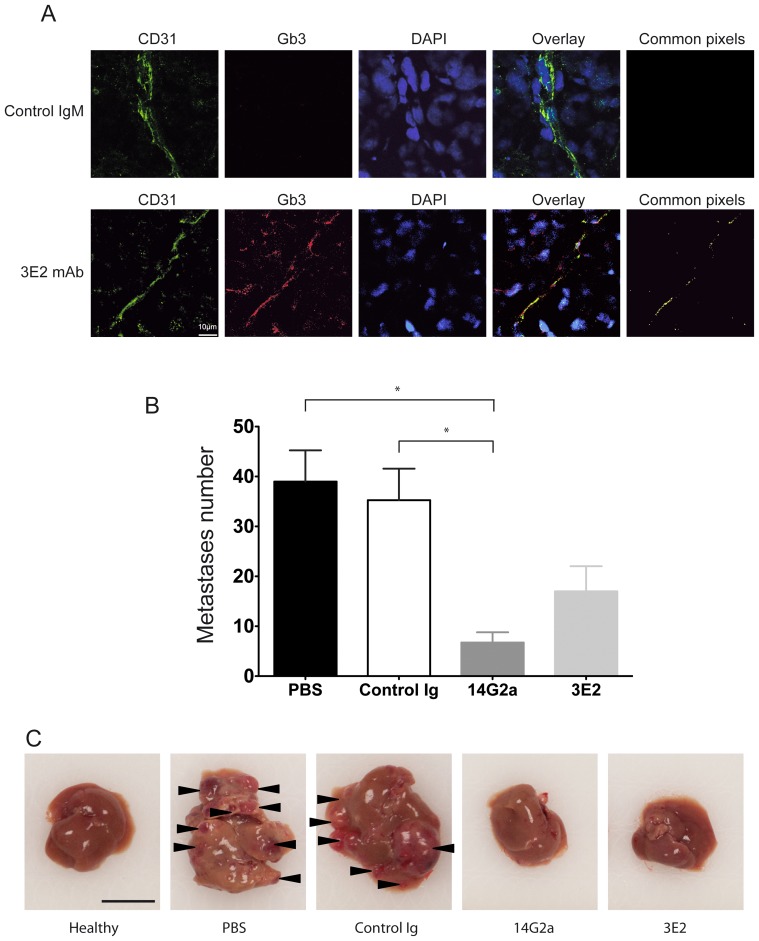
3E2 inhibits in *vivo* metastases spreading. A) Pictures of NXS2 hepatic metastases by confocal microscopy stained with Alexa488-conjuguated CD31 mAb (green), Alexa568-conjuguated 3E2 (red) or isotypic IgM control and counterstained with Draq5. Colocalization of the endothelial marker CD31 and Gb3 is shown by the yellow staining on the merge image. B) Number of liver metastases per animal (n = 6; mean±SEM; *p<0.05). C) Representative photography of liver 28 days after NXS2 injection and the different immunotherapies (scale bar represents 1 cm).

Because 3E2's role in inhibiting *in vitro* cell proliferation, we further investigated whether 3E2 specifically targets blood vessels within the tumor mass. We first developed 3E2 for an immunohistological approach (Fig. S3). After 3E2 hybridization, a strong brown DAB peroxidase staining was observed in Gb3-positive RAJI tumor sections, but not in Gb3-negative IMR32 tumor sections. Because of its potential anti-angiogenic drug usable *in vivo*, we decide to determine the therapeutic efficacy of 3E2 in Gb3-negative metastases tumor model after injection of neuroblastoma NXS2 cells in the tail vein [20]. The validation of the Gb3 status of the tumor model and the tumor vessel were obtained by the staining for Alexa 568-conjugated 3E2 (red) and Alexa 488-conjugated CD31 (green) in NXS2 metastases frozen sections, which revealed a colocalization of Gb3 and endothelial marker as shown by the yellow staining on the merged image (Fig. 6A). To determine the anti-tumor efficacy of 3E2, the number of NXS2 liver metastases was counted 28 days after tumor cell injection. A positive responder cohort was established using mAb 14G2a directed against the neuroblastoma associated tumor antigen disialoganglioside GD2 (Fig. 6B and C). Both treatments were effective in reducing liver metastases, as indicated by a decrease of the number of liver metastases from 35±6.3 in control mice to 17±5.0 in 3E2-treated mice or 6.7±2.1 in 14G2a-treated mice (both p<0.05 vs. control). The number of metastases after 3E2 and 14G2a treatments was neither statistically different against each other nor against healthy control animals (both p>0.05). Finally, the distribution of Gb3 in mouse healthy tissues was evaluated by immunohistology (Table 1). In summary, 3E2 binds only Gb3 present in kidney, skin, eye and testis. Most normal tissues did not express any detectable amount of Gb3. Because 3E2 is an IgM which distribution is limited to the intravascular compartment and because it recognizes blood vessels in solid tumor, but not vessels from healthy tissues, our results suggest that its therapeutics effect is likely to be restricted to these vessels.

**Table 1:** Distribution of Gb3 in organs from C57Bl/6 mouse obtained after staining with Alexa568-conjuguated 3E2.

Tissue	Gb3	Isotypic control
Abdominal fat	−	−
Brain	−	−
Colon	−*	−*
Duodenum	−	−
Esophagus	−	−
Eyes	+	−
Heart	−	−
Kidney	+	−
Liver	#	#
Lung	−	−
Muscles	−	−
Ovaries	−	−
Pancreas	−	−
Pleura	−	−
Skin	+	−
Spleen	−	−
Stomach	−	−
Testis	+	−
Thyroid	−	−
Uterus	−	−

Frozen healthy organs sections of 5 µm were hybridized with biotinylated 3E2 or its isotypic control, revealed by an Alexa 568-conjugated streptavidin and counterstained with DAPI. Pictures were observed under a confocal microscope (n = 3).

# : High background.

* : Lipofuscin autofluorescence.

### 3E2 mitigates the growth of NXS2 established tumors

**Figure 7:**
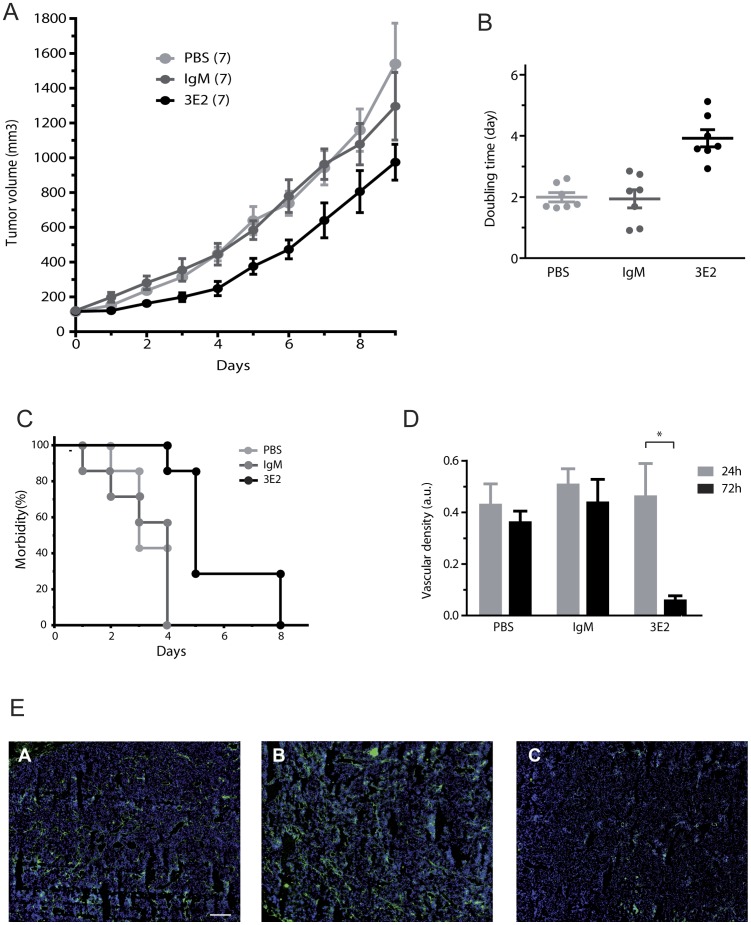
3E2 mitigates tumor growth. A) Tumor volume of NXS2 tumors (n = 7; mean±SEM; *p<0.05). B) Doubling time of the each tumors (•) estimated during the four first days of the different treatments (bars: mean±SEM; *p<0.01; n = 7). C) Actuarial Morbidity curves of to tumor reaching 300 mm^3^ (n = 7). D) Vascular density estimated on 50 different slides on 3 different treated tumors (mean±SEM; *p<0.01; n = 3). E) Mosaic pictures by apotome microscopy stained with Alexa488-conjuguated CD31 mAb (green) and counterstained with Draq5 (bar: 500 µm).

Because anti-angiogenic drugs are also mitigating tumor development, we next evaluated the anti-tumor effects of anti-Gb3 mAb 3E2 in A/J mice with subcutaneous established NXS2 tumors. First, we measured daily the tumor volume after two injections of either 3E2- or IgM control- antibody (500 µg) at day 0 and 3 in 100 mm^3^-established tumors. The anti-Gb3 mAb treatment strongly delayed the development of the fast growing NXS2 tumors (p<0.05 between day 2 and 7 as compared to PBS- or IgM-treated tumors; Fig. 7A). In fact, the tumor growth was diminished by 3E2 treatment. The time for the tumors to reach 300 mm^3^ was delayed in 3E2-treated group (median morbidity for 3E2 5 days vs. 3 for PBS and 4 for IgM; p<0.01 Fig. 7B). This result was in correlation with a rise by two fold of the volume doubling time in the mice treated with anti-Gb3 antibody (mean doubling for 3.2-treated tumors 3.92 days vs. 1.99 and 1.94 for respectively PBS- and IgM-treated tumors, p<0.01; Fig. 7C). Finally, we measured the blood vessel density at day 1 and day 3. No difference in the blood vessel density was observed at day 1. On the other hand, a decrease of 70% of blood density was observed in tumors from the mice that had received mAb 3E2 compared to the IgM-control group (p<0.1; Fig. 7D and E). Taken altogether, these data evidence an action of anti-Gb3 mAb 3E2 on the NXS2 tumor vasculature.

## Discussion

Overall, our findings show that the mouse mAb IgM 3E2 specific for globotriaosylceramide Gb3, over-expressed in proliferating endothelial cells, displays anti-angiogenic properties, including *in vitro* modulation of the viability, inhibition of proliferating endothelial cells, *ex vivo* inhibition of endothelial cell sprouting in mouse aorta ring assay, and *in vivo* tumor growth suppression.

Although GSLs are able to modulate angiogenesis [32], the exact glycolipid composition of the human endothelial cells has generally received limited attention. Immunological detection of Gb3 using specific mAbs or verotoxins, in combination with anti-verotoxin antibodies, has demonstrated that Gb3 represent the dominant glycolipids among Gb4 in human umbilical endothelial cells (HUVECs) [33], [34]. Gb3 and Gb4 are also the major glycolipid in primary and immortalized human brain microvascular endothelial cells (HBMECs) [35]. In addition, previous observations suggest that inflammatory mediators, including TNFα, IFNγ and LPS, up-regulate Gb3 level in the endothelial cells [36]. In this report, we further demonstrated that Gb3 concentration is increased in proliferating endothelial cells, in the presence of growth factors (complete medium) or pro-angiogenic factor S1P [37]. Our data suggest that endothelial cells require additional signals including pro-angiogenic molecules such as S1P to induce Gb3 synthesis. They also question whether Gb3 might play a role in the endothelial cell phenotypic changes during angiogenesis, as it was shown for its precursor LacCer in VEGF-induced angiogenesis [38]. Yet, our data, which are in agreement with others [39], point to Gb3 as an angiogenic marker for developing anti-angiogenic vectors. However, Gb3 is certainly not the only lock limiting step in angiogenesis and tumor development. The understanding of the connection between the different pro-angiogenic molecular pathways remains important to better define the adequate anti-angiogenic treatment using a single or a couple of concomitant drugs.

Because of the crucial issues regarding the immunogenicity of verotoxin-based vector, however, we sought to generate a mouse mAb specific for Gb3. We therefore immunized Balb/c mice with proliferating primary HMVEC cells activated by colic T84 adenocarcinoma cells, which are not expressing Gb3 (Fig. 2B and [40]) in a co-culture system. After screening on angiogenic endothelial cell membranes, 6 hybridoma clones were selected against Gb3. Antibody 3E2, an IgM-class mAb, was chosen for its strong binding properties. If the majority of therapeutic antibodies derived from IgG-class, IgM-class antibodies specific to glycolipid antigens offer unique advantages that make them highly desirable as cancer therapeutics. For example, IgM molecules are decavalent antibodies with high avidity for the target antigen. Their high valences also facilitate cell surface receptor cross-linking which in turn may lead to a more effective cell killing [41]. In patients with metastatic melanoma, GM3 ganglioside is more effectively targeted by IgMs than it is by IgGs, [42]. Furthermore, in the case of Gb3, a potential advantage of using an IgM-class antibody is that its tissue distribution is generally limited to the vascular compartment due to their large molecular weight. Thus, normal Gb3-expressing cells would not be negatively affected by hostile IgM antibodies.

For cancer immunotherapy, specificity of the antibody including binding affinity, CDC, ADCC, and signaling activity are critical. As for most of the IgM-class antibodies, 3E2 was efficient in inducing potent CDC-, but not ADCC-, activity against Gb3-expressing endothelial cells (Fig. S4) [43]. Interestingly, we also showed that 3E2 inhibits directly high Gb3-expressing cycling endothelial cell proliferation and subsequently angiogenesis offering a cytostatic property to our anti-Gb3 mAb. Moreover, Gb3 or Gb3 synthase expressions were not modify by mAb 3E2 treatment, which provide the opportunity to use sequentially our antibody for prolong anti-tumor effect. Despite previous reports showing on B cells [44] or on Burkitt's lymphoma cells [45] an apoptosis induced by anti-Gb3 mAbs or shiga toxin B subunit, any increase of endothelial cell death was observed by time lapse or Hoechst staining or sub-G1 analysis after incubation of HMEC-1 with 40 µg/ml of 3E2 (Fig. 5E). However, apoptosis-like death driven by anti-Gb3 mAbs is not considered as a common feature of all Gb3-reacting mAbs [46] and might be dependent of the Gb3 species recognized by the antibody. In fact, fatty acid heterogenicity [47], hydroxylation [48], and/or chain length and unsaturation degree [49] may influence the lateral mobility of Gb3 in the plasma membrane, as well as the conformation of the trisaccharide head group presented at the cell surface. In the same manner, the structure of glycolipid ceramide moieties influences the binding activity of antibody [50]. Although the exact roles of the ceramide heterogenicity of Gb3 in antibody inhibitory signaling functions remain unclear, we hypothesize a functional role of certain Gb3 species that might provide a molecular basis for the different anti-Gb3 mAbs inhibitory effects.

Finally, we reported for the first time that passive immunotherapy with anti-Gb3 mAb 3E2 is effective in suppressing the growth of tumor and the spreading of metastases in murine syngeneic neuroblastoma models, which is relevant to the human disease [20]. Those models expresses GD2, a well-established tumor-associated neuroblastoma antigen [20], but not Gb3 as demonstrated here *in vitro* and *in vivo*. The distribution of Gb3 within the NXS2 metastases, evidenced by 3E2 overlay and validated via CD31 staining, is clearly limited to the blood vessel endothelial cells of the tumor compartment. These findings are consistent with previous observations of verotoxin binding to the neovasculature of frozen human neuroblastoma sections without any cross-reactivity with the tumor cells themselves [13], [15], [39], [51], and demonstrate the relevance of our model. Yet, because Gb3 expression was limited to the tumor microvasculature, our result demonstrated that anti-vascular activity mediated by anti-Gb3 mAbs, is sufficient to inhibit blood vessels development (Fig. 7D and E), the growth of transplanted tumors (Fig. 7A) and the spreading of metastases (Fig. 6B). They also demonstrate that the anti-tumor efficacy of anti-Gb3 mAb 3E2 is comparable to that of anti-GD2 mAb 14G2a, which targets directly NXS2 neuroblastoma and has undergone clinical evaluation after positive results [52]. Interestingly, previous studies implicate angiogenesis as an essential mechanism regulating neuroblastoma growth [53] emphasizing the beneficial effect of mAbs specific for Gb3 in anti-angiogenesis treatment strategies in neuroblastoma.

Renal microvascular endothelium and kidney epithelia express Gb3 and might therefore be negatively affected by anti-Gb3 binding compounds [54]. In particular, the apoptotic effect of verotoxin may lead to severe side-effects due to the targeting of normal Gb3-expressing cells outside the tumor vasculature, especially the microvasculature of the kidney [55]. Potential molecular targets for mAb-mediated anti-angiogenic immunotherapy should be expressed in a specific-tumor endothelial cell manner, unless the normal cells are not affected by antibody binding. As mentioned above, binding of 3E2 to Gb3 induces inhibitory signal potentially linked to AKT and ERK activation, in proliferating endothelial cells independently of immune effectors and apoptosis. Yet, the inhibitory effects of 3E2 to endothelial cell proliferation and angiogenesis independently of immune effectors recruitment and apoptosis, is particularly interesting because it may represent a novel strategy in limiting toxic verotoxin effects *in vivo*. In fact, no gross toxicity in mice was observed when 3E2 was injected. By Fc-engineering, it is possible to modulate the CDC activity of mAbs [56]. Therefore, the development of anti-Gb3 immunotherapeutic agents with decreased complement-dependent lyses may offer treatment options with reduced adverse side effects on the normal Gb3-expressing cells.

In conclusion, the mAb 3E2 induced inhibition of tumor-induced angiogenesis, coupled with the increased level of Gb3 found in the tumor cells and metastases indicate that anti-Gb3 treatment offers a promising alternative for verotoxin-based Gb3 ligands. Our observations further suggest the possibility for engineering new Gb3 specific vectors with limited toxicity to normal Gb3-expressing quiescent cells. Further investigations to determine and compare signaling pathways used by endothelial cells after Gb3 engagement by specific mAbs leading to anti-angiogenic activities are warranted.

## Supporting Information

Figure S1 **Glycolipids staining by orcinol after HPTLC shown a Gb3 expression in HMEC-1 and RAJI, but not in NXS2.** Left panel lane 1: standard rat brain gangliosides, lane 2: standard neutral GSLs, lane 3: puri•ed Gb3, lane 4: HMEC-1 glycolipids extract. Right panel glycolipids extract from: lane 1, HMEC-1; lane 2, RAJI; lane 3: NXS2.
(PDF)

Figure S2 **Increased division time in 3E2-treated endothelial cells.** Photographs of HMEC-1 cell division after IgM or 3E2 treatment using 20× magnification. Arrow heads shows dividing cells.
(PDF)

Figure S3 **Immunohistology of Gb3-positive tumors in Balb/c mice by 3E2.** Gb3-positive RAJI tumor slices hybridized with 40 µg/ml 3E2, and then reveled by the brown staining of DAB-peroxidase (upper left). Control IgM (upper right) and Gb3-negative IMR32 tumors (lower right and left) showed no staining. Nuclei were counter-stained with hematoxilin (blue). Magnification 40×.
(PDF)

Figure S4 **3E2 induces a complement dependent cytotoxicity in HMEC-1 and RAJI Gb3-positive cells, but not in NXS2 Gb3-negative cells.** CDC was measured as the % of cell lysis induced by the 10 µg/ml of 3E2 with human serum for 2 h. Specifc lysis was determined by Facs using propidium iodide (n = 3; mean±SEM; *p<0,05).
(PDF)
