# Supplementary material for: Anti-Gb3 Monoclonal Antibody Inhibits Angiogenesis and Tumor Development
Source: PLoS One. 2012 Nov 26;7(11):e45423. doi: 10.1371/journal.pone.0045423 (PMC3506626; doi:10.1371/journal.pone.0045423)
Supplement: Figure S1 — Glycolipids staining by orcinol after HPTLC shown a Gb3 expression in HMEC-1 and RAJI, but not in NXS2. Left panel lane 1: standard rat brain gangliosides, lane 2: standard neutral GSLs, lane 3: puri•ed Gb3, lane 4: HMEC-1 glycolipids extract. Right panel glycolipids extract from: lane 1, HMEC-1; lane 2, RAJI; lane 3: NXS2. (PDF) [file pone.0045423.s001.pdf]

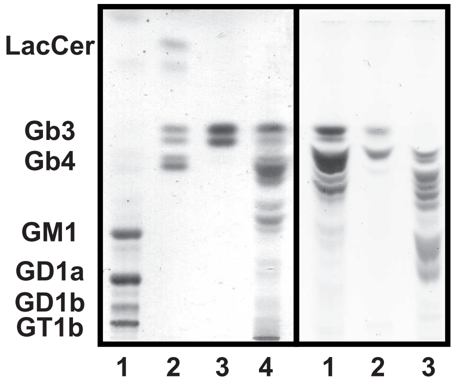

Figure S1: Glycolipids staining by orcinol after HPTLC shown a Gb3 expression in HMEC-1 and RAJI, but not in NXS2. Left panel lane 1: standard rat brain gangliosides, lane 2: standard neutral GSLs, lane 3: purified Gb3, lane 4: HMEC-1 glycolipids extract. Right panel glycolipids extract from: lane 1, HMEC-1; lane 2, RAJI; lane 3: NXS2.
