# Supplementary material for: Anti-Gb3 Monoclonal Antibody Inhibits Angiogenesis and Tumor Development
Source: PLoS One. 2012 Nov 26;7(11):e45423. doi: 10.1371/journal.pone.0045423 (PMC3506626; doi:10.1371/journal.pone.0045423)
Supplement: Figure S2 — Increased division time in 3E2-treated endothelial cells. Photographs of HMEC-1 cell division after IgM or 3E2 treatment using 20× magnification. Arrow heads shows dividing cells. (PDF) [file pone.0045423.s002.pdf]

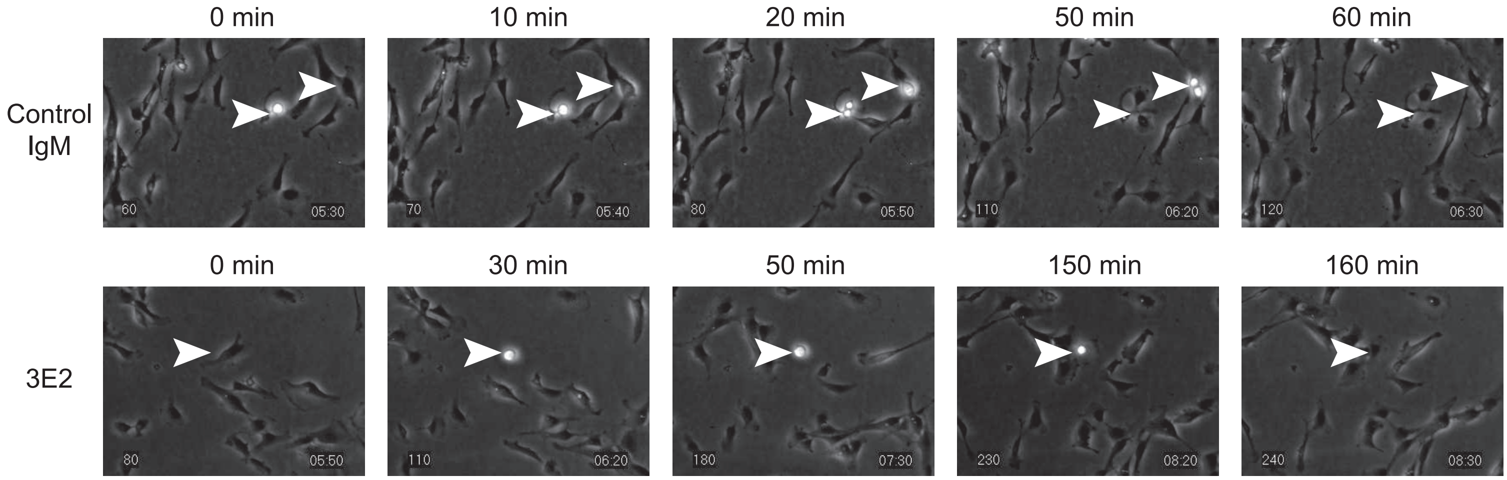

Figure S2: Increased division time in 3E2-treated endothelial cells. Photographs of HMEC-1 cell division after IgM or 3E2 treatment using 20x magnification. Arrow heads shows dividing cells.
