# Supplementary material for: Anti-Gb3 Monoclonal Antibody Inhibits Angiogenesis and Tumor Development
Source: PLoS One. 2012 Nov 26;7(11):e45423. doi: 10.1371/journal.pone.0045423 (PMC3506626; doi:10.1371/journal.pone.0045423)
Supplement: Figure S3 — Immunohistology of Gb3-positive tumors in Balb/c mice by 3E2. Gb3-positive RAJI tumor slices hybridized with 40 µg/ml 3E2, and then reveled by the brown staining of DAB-peroxidase (upper left). Control IgM (upper right) and Gb3-negative IMR32 tumors (lower right and left) showed no staining. Nuclei were counter-stained with hematoxilin (blue). Magnification 40×. (PDF) [file pone.0045423.s003.pdf]

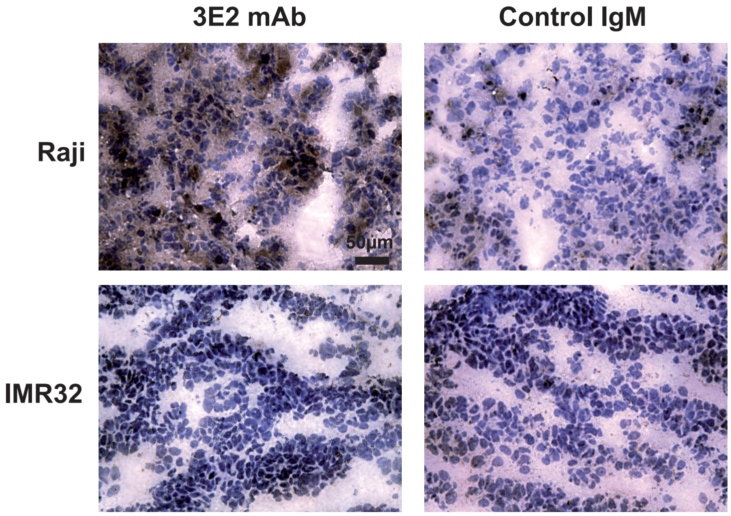

Figure S3: Immunohistology of Gb3-positive tumors in Balb/c mice by 3E2. Gb3-positive RAJI tumor slices hybridized with 40µg/ml 3E2, and then reveled by the brown staining of DAB-peroxidase (upper left). Control IgM (upper right) and Gb3-negative IMR32 tumors (lower right and left) showed no staining. Nuclei were counter-stained with hematoxilin (blue). Magnification 40x.
