# Supplementary material for: Anti-Gb3 Monoclonal Antibody Inhibits Angiogenesis and Tumor Development
Source: PLoS One. 2012 Nov 26;7(11):e45423. doi: 10.1371/journal.pone.0045423 (PMC3506626; doi:10.1371/journal.pone.0045423)
Supplement: Figure S4 — 3E2 induces a complement dependent cytotoxicity in HMEC-1 and RAJI Gb3-positive cells, but not in NXS2 Gb3-negative cells. CDC was measured as the % of cell lysis induced by the 10 µg/ml of 3E2 with human serum for 2 h. Specifc lysis was determined by Facs using propidium iodide (n = 3; mean±SEM; *p<0,05). (PDF) [file pone.0045423.s004.pdf]

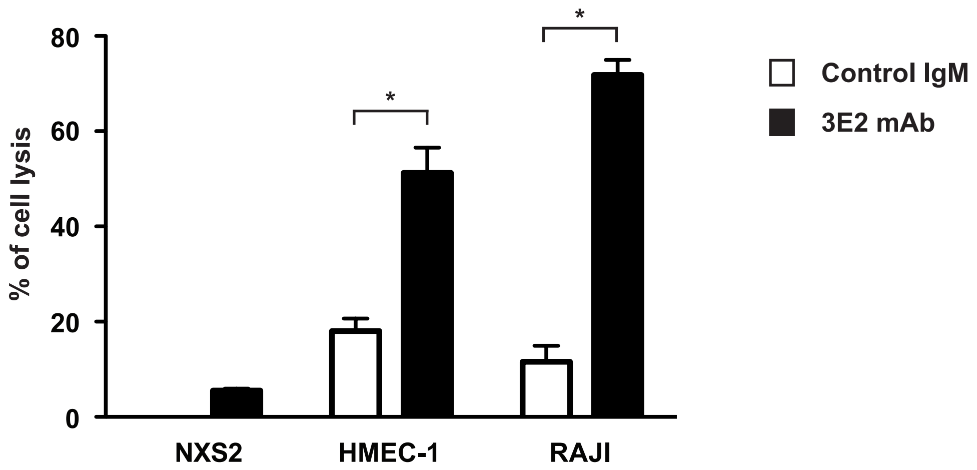

Figure S4: 3E2 induces a complement dependent cytotoxicity in HMEC-1 and RAJI Gb3-positive cells, but not in NXS2 Gb3-negative cells. CDC was measured as the % of cell lysis induced by the 10 μg/ml of 3E2 with human serum for 2h. Specific lysis was determined by Facs using propidium iodide (n=3; mean±SEM; *p<0,05).
